# Supplementary figures and images for: Transgenerational Cold Acclimation and Contribution of Gut Bacteria in Spodoptera frugiperda
Source: Insects. 2025 Oct 16;16(10):1052. doi: 10.3390/insects16101052 (PMC12564332; doi:10.3390/insects16101052)

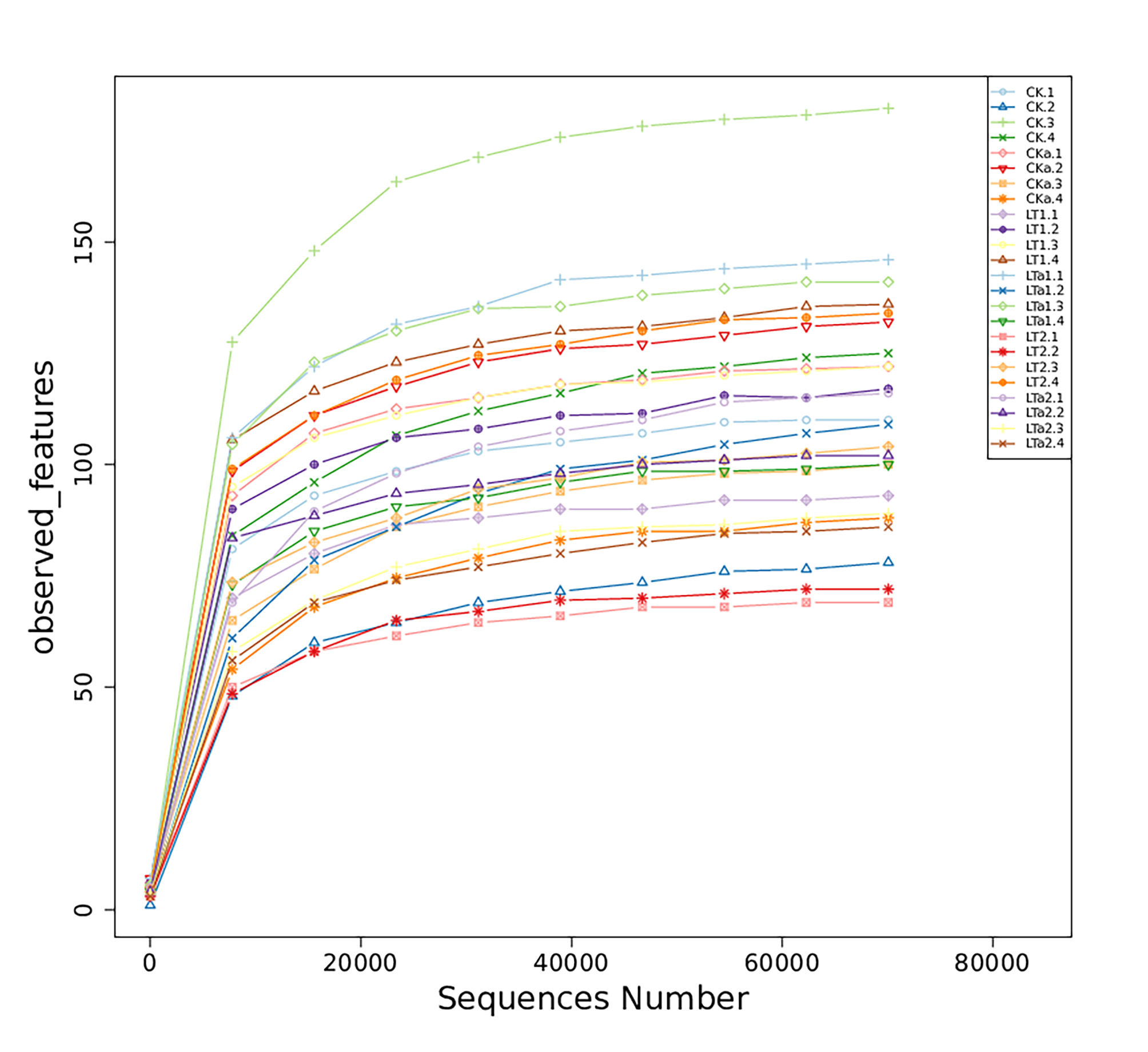

Supplement: Supplementary file 1 [file insects-16-01052-s001.zip › Figure S1.tif]
